# Exercise in the Elderly from the Perspective of Persian Medicine: A Solution to Prevent Chronic Diseases

**DOI:** 10.31661/gmj.v9i0.1742

**Published:** 2020-11-26

**Authors:** Marzieh Alazadeh, Fatemeh Niksolat, Seyde Sedighe Yousefi

**Affiliations:** ^1^Faculty of Medicine, Mazandaran University of Medical Sciences, Sari, Iran; ^2^Student Research Committee, Traditional and Complementary Medicine Research Center, Addiction Institute, Mazandaran University of Medical Sciences, Sari, Iran; ^3^Orthopedic Research Center, Faculty of Medicine, Department of Internal Medicine, Mazandaran University of Medical Sciences, Sari, Iran

**Keywords:** Exercise, Elderly, Persian Medicine, Chronic Diseases

## Dear Editor,

Chronic diseases are distinguished in terms of the public health care system because chronic diseases may cause disability and impose a high cost per year on society. The elderly is often associated with several chronic conditions such as cardiovascular, osteoarthritis, depression, cancer, diabetes, and mental disorders. One of the best practices for preventing chronic diseases is exercise. There is a relationship between increased obesity and the prevalence of chronic diseases [1]. The prevalence of obesity has increased in recent decades, and given that obesity has a significant role in the failure of the treatment of chronic diseases [2], this issue has become more important. It is important to find a solution that can affect many chronic diseases and obesity problems. The first step toward the treatment of many chronic diseases is non-drug therapy, such as exercise. Today, considering the immobility in life, exercise is a fundamental requirement. The waste substances are constantly accumulated in the body, and the best solution for reducing these substances is daily exercise [3]. Exercise is cheap, and it can play an essential role in the health of individuals. It should be noted that increasing the prevalence of diseases in the old ages is partly due to the reduced function in the immune system [4], and moderate exercise can improve immunity [5]. Persian medicine is one of the oldest medical systems. In Persian medicine, one of the principles of maintaining health is movement, and exercise is a movement that is called “ *Riazat*” in its literature [6]. *Riazat* is a voluntary movement in which intensive and more frequency inhalation and exhalation are carried out. In a classification, exercise is classified as a general and particular type. General exercise improves the health of the whole body, such as walking and horseback riding, but in a particular exercise specific to an organ, which benefits more, such as stretching the limbs [7]. In terms of Persian medicine, an appropriate exercise for the elderly is walking. The importance of exercise in aging is to the extent that recommended; if a person cannot walk for some reason, the use of a hammock is the best option [6]. Exercising is one of the basic principles of maintaining health conditions, but one should consider factors such as not indulging in exercising, and it is also better to do a variety of exercises, not a single exercise [7]. In order to benefit from the effects of exercise, one should pay attention to the followings: (1) considering the physical condition of an individual, in terms of strength and weakness; the type of exercise should be proportional to the individual’s physical strength, (2) the type of disease, in terms of acute or chronic and the affected organ, (3) age, (4) environmental conditions [8], and (5) the intensity of exercise. As shown in Table-1, exercise is classified into mild, moderate, and intense.

In the mild exercise, the skin feels subtle heat, and in the medium, warmth and redness of the skin become more, and the body sweats up a little, and in the intensive exercise, warmth and redness of the skin become very, and the body is very sweating [7]. The exercise should be moderate in terms of health maintenance and disease prevention. Since older people often do not have a good function, moderate exercise is recommended to them. The intensive exercise can cause damage to the body of the elderly [9]. However, the intensity of exercise should be individualized based on the elderly health condition. Intensive exercise can also lower body immunity, while moderate exercise can increase one’s immune system and improve mental conditions [10]. Humidity should be moderate, and the place of exercise should be equipped with air conditioners. Time and season are necessary; indeed, it is better to exercise when the temperature is more moderate than the other hours.

## Acknowledgment

 There was no financial support for this study.

## Conflict of Interest

 The authors declare that there is no conflict of interest.

**Table 1:** Environmental Conditions and How to Exercise

**Seasons**	**Time**	**Duration**	**Intensity**
Spring	Near noon	Long-time	Moderate
Summer	Morning	Short-time	Weak
Autumn	Near noon	Brief	Moderate
Winter	Evening	Long-time	Intense
